# Robust Stride Segmentation of Inertial Signals Based on Local Cyclicity Estimation

**DOI:** 10.3390/s18041091

**Published:** 2018-04-04

**Authors:** Sebastijan Šprager, Matjaž B. Jurič

**Affiliations:** Faculty of Computer and Information Science, University of Ljubljana, Večna pot 113, SI-1000 Ljubljana, Slovenia; matjaz.juric@fri.uni-lj.si

**Keywords:** inertial sensors, stride segmentation, gait assessment, inertial signals, biomedical signal processing

## Abstract

A novel approach for stride segmentation, gait sequence extraction, and gait event detection for inertial signals is presented. The approach operates by combining different local cyclicity estimators and sensor channels, and can additionally employ a priori knowledge on the fiducial points of gait events. The approach is universal as it can work on signals acquired by different inertial measurement unit (IMU) sensor types, is template-free, and operates unsupervised. A thorough evaluation was performed with two datasets: our own collected FRIgait dataset available for open use, containing long-term inertial measurements collected from 57 subjects using smartphones within the span of more than one year, and an FAU eGait dataset containing inertial data from shoe-mounted sensors collected from three cohorts of subjects: healthy, geriatric, and Parkinson’s disease patients. The evaluation was performed in controlled and uncontrolled conditions. When compared to the ground truth of the labelled FRIgait and eGait datasets, the results of our evaluation revealed the high robustness, efficiency (F-measure of about 98%), and accuracy (mean absolute error MAE in about the range of one sample) of the proposed approach. Based on these results, we conclude that the proposed approach shows great potential for its applicability in procedures and algorithms for movement analysis.

## 1. Introduction

Efficient and robust stride segmentation is a well-known procedure that has become widely applicable with the appearance of novel measuring principles for human motion, and is fundamental for the related analysis procedures. Stride segmentation is becoming increasingly attractive with respect to the development of the IoT (Internet of things) paradigm [1]. Contemporary measuring principles are based on ubiquitous and pervasive sensing and computing by leveraging service-oriented and cloud architectures that enable remote and continuous monitoring of human motion data collected by sensing devices during daily living. Thus, the IoT paradigm has enabled the transition of the monitoring, observation, and analysis principles of human motion from controlled environments in the areas of biomedicine and biomechanics, to daily life. This has been achieved by the introduction and development of novel advanced approaches, especially in the fields of telemedicine, neurorehabilitation, sports, fitness, biometry, navigation, localization, and many others that enable and fully support the realization of human motion analysis tasks in everyday environments [2,3,4,5].

Motion data during gait is collected in the form of biomedical signals that can be obtained by a variety of sensing devices capable of detecting motion dynamics, usually in terms of motion trajectories. Wearable sensors [6] are very attractive and fully compliant with the IoT paradigm. These sensors measure the motion dynamics of a specific part of human body according to where the sensing device is attached. Motion dynamics are interpreted by instantaneous accelerations measured by accelerometers, angular speed by gyroscopes, and magnetic field by magnetometers. Since they are most commonly implemented as MEMS, they are attractive and widely used, especially due to their light weight, low cost, small size, and low energy consumption. In fact, nowadays they are integrated in practically every smart device, thus making inertial/magnetic data-based processing approaches widely applicable. Furthermore, inertial/magnetic sensors have already been established by thorough gait analysis in the clinical environment by novel approaches [7,8], where efficient and robust stride segmentation is of great importance [9,10].

The majority of successful approaches for gait analysis rely on the observation of particular strides. A single stride represents an elementary part of a gait sequence and can be defined as single gait cycle [11]. Stride time is the time interval between two successive occurrences of gait cycles. Each stride can be interpreted by fiducial points—time instants that determine specific gait event or phase (i.e., heel strike, toe-off, mid-stance, etc.). Each gait sequence consists of consecutive strides that are represented by signals corresponding to measured physical quantity (i.e., acceleration). Gait properties are reflected through the morphology of strides that is described by motion dynamics as observed in the sensing point of the particular part of human body. Due to the cyclical property of the gait it can be inferred that the morphology of strides will be similar for a particular subject and its stride in specific conditions [12], where gait-affecting factors (i.e., health, mood, surface, etc.) can significantly influence the morphology of strides. During gait analysis, a proper declaration of gait events and phases is also of particular importance. Consequentially, through a proper interpretation of stride morphology and gait events, the main task of gait analysis is to extract specific gait parameters. At this point it is important to emphasize that the performance of practically all recent gait analysis approaches is highly dependent on the performance of stride segmentation. Thus, it plays a crucial role in many gait-related problems in several areas of application and research, from simple (step counter [13,14,15], gait frequency estimation) to advanced i.e., physiological gait parameter estimation [16] (step length [17,18,19], gait speed [20,21,22], 6MWT [23], etc.), gait-based authentication [12], navigation [5], localization [4], human characteristics [24], activity [14], and gait action [25], among others.

### Related Work

Gait segmentation approaches can be categorized according to operation principle, employed sensor type, stride treatment, and applicability mode. With respect to the operation principle, due to their simplicity and low computational demands, peak-based approaches are amongst the most popular and are usually extended with special enhancements in order to improve robustness, i.e., special filtering, zero-crossing detection, adaptive thresholding, etc. [4,5,15,23,24,25,26]. Peak-based approaches are suitable in the situations where precise segmentation of individual strides is not crucial. Therefore, the most recent relevant approaches rely on gait morphology, including machine learning and pattern recognition approaches such as dynamic time warping, the hidden Markov model, k-means clustering, wavelet transformation, etc. [9,10,13,21,27].

From the perspective of usability, inertial/magnetic sensor types that are employed for stride segmentation can be divided into two groups: special-purpose sensors and multi-purpose sensors. Special-purpose sensors are constructed so that they are compliant to the problem they are solving by measurement and data processing, usually directly related to the field of application, i.e., biomedicine, neurorehabilitation, etc. These sensors are implemented as special evaluation boards and are directly mounted on specific body positions, either directly or indirectly (i.e., shoe sensors). They are applied for more accurate estimation of gait parameters. Therefore, they demand fixed mounting and higher sampling frequencies and resolution, resulting in stable, descriptive, and low-noise gait patterns within acquired inertial/magnetic signals. The corresponding relevant stride segmentation approaches are compliant with these properties [9,10,21,25]. In contrast, the multi-purpose sensors that are nowadays integrated in smart devices (phones, tablets) are subject to causal and relaxed use, and inertial/magnetic signals are sampled with low sampling frequency and lower resolution. Thus, the majority of existing approaches are focused more on simple detection of strides (step counting), while accurate positioning of strides (in terms of gait events) is far more challenging [4,13,15,26,27]. Finally, the appearance of commercial activity sensor monitors should not be overlooked, as one of the most important functionalities of these commercially available physical monitors for activity recognition is also step detection—some of the most recent monitors have been evaluated by Strom et al. [14].

Individual strides represent a basis for stride segmentation approaches. According to the accuracy of stride treatment, related approaches can be divided into two groups: simple and advanced. The first group covers approaches that are considered sufficient if particular strides are simply detected and the precision of its position is not the biggest concern. Such approaches are feasible for implementation of step counters and simpler gait assessment procedures [4,13,26,27]. Conversely, accurate stride segmentation is needed for better description of individual stride, usually in terms of gait events needed for further procedures for thorough gait analysis [9,10,16,21,25].

As a result our recent research activities, we provide the following outcomes and scientific contributions: (1) an introduction and evaluation of a robust approach for stride segmentation, gait sequence extraction, and accurate assessment of gait events; and (2) presentation of a FRIgait dataset that contains inertial/magnetic data acquired by smart devices during a free walk meant to investigate and evaluate gait assessment procedures in realistic and uncontrolled conditions. Labelled inertial/magnetic gait data collected by smartphones in daily life is of great importance when developing and evaluating approaches for advanced gait analysis in uncontrollable scenarios. Existing datasets, i.e., the largest available dataset proposed by Ngo et al. [28], are mainly focused on short-term measurements in controlled conditions.

When compared with the most relevant approaches, the proposed approach reveals the following advantages: (1) multi-method, multi-modal, and multi-sensor operation capability; (2) unsupervised operability that does not apply any training procedure (as employed by learning-based approaches similar to those published in recent works of [29,30]), nor any prior knowledge on the shape/morphology of strides (templates); (3) sufficient consideration of time-variance in stride shapes (transition phases); (4) accurate estimation of stride times; (5) accurate positioning of gait events; (6) reliable extraction of gait sequences; and (7) flexible and modular operation capability. Performance of the proposed approach in terms of robustness, efficiency, and accuracy was evaluated on FRIgait and eGait datasets with the following aspects in order to address the research hypotheses: gait sequence extraction performance, stride segmentation performance, and stride positioning accuracy.

## 2. Methodology

It is well known that gait is represented as a cyclostationary process [12]. Related work points out that the majority of existing IMU-based approaches for stride segmentation are focused on the detection of individual cyclic events that appear in inertial signals, either on a peak detection or on a morphological basis. In contrast, the proposed method is based on the concept of observing local cyclicity through varying fundamental frequencies in observed inertial signals (pitch tracking). More precisely, the proposed approach is based on the estimation of instantaneous fundamental frequency in terms of interval length between two consecutive strides (stride time). It is based on the similarity between consecutive strides, followed by reconstruction of exact stride positions (time instants). One of the crucial parts of the approach was inspired by interval length estimation procedure proposed by Brüser et al. [31,32]. Their procedure was efficiently applied for estimation of inter-beat intervals in ballistocardiographic signals.

The adapted core procedure is based on the processing of sequential analysis windows that run through inertial/magnetic signals sampled by sampling frequency $f_{\mathrm{samp}}$. The analysis window should cover at least two strides upon which an instantaneous fundamental frequency is estimated. The estimation procedure is based on an a priori knowledge on the boundaries of the interval corresponding to fundamental frequencies of observed phenomena (stride), determined by $f_{\max}$ and $f_{\min}$, respectively standing for the maximum and minimum expected stride frequency. The corresponding admissible maximum and minimum stride times are defined as $N_{\max}=\frac{f_{\mathrm{samp}}}{f_{\min}}$ and $N_{\min}=\frac{f_{\mathrm{samp}}}{f_{\max}}$, respectively. These admissible interval lengths are then considered within the stride time estimation procedure for each position of the analysis window. Analysis window positions are determined by a fixed, sufficiently small step η. In this manner, a redundancy by multiple sequential estimation of the same interval is obtained which is reflected in the robustness of the proposed approach.

**Figure 1:**
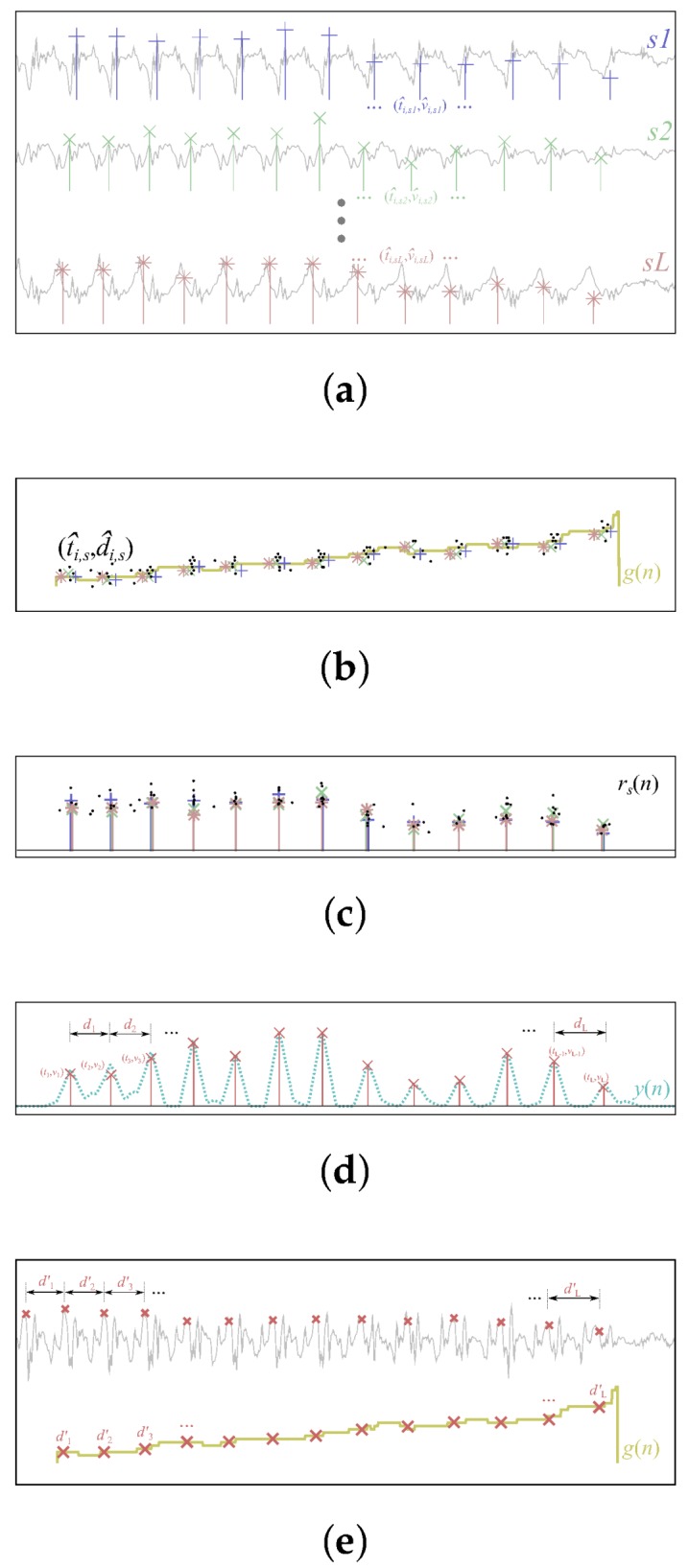
Pipeline of the proposed stride segmentation approach. (**a**) Component-wise local cyclicity estimation; (**b**) Assessment of stride positions; (**c**) Alignment of assessed stride positions; (**d**) Fusion of aligned stride positions; (**e**) Enhancement of assessed stride positions on the reference component.

Let *X* represent a compound inertial signal of length *ℓ* collected by measurements during the observation of the same phenomena with sampling frequency $f_{\mathrm{samp}}$:

(1) $$X\left(n,s\right)=\left[x_{s_{1}}\left(n\right)\ldots x_{s_{L}}\left(n\right)\right]$$

where $s\in \{s_{1}\ldots s_{L}\}$ represents sensing modalities. i.e., in the case of a single sensing device compound of an accelerometer, gyroscope, and magnetometer, and X(n,s) has nine components (sensor channels) if all three sensors are 3-axial. Variable *s* can also denote components from multiple sensing devices, virtual sensors, or transformed quantities (i.e., accelerometer magnitude). The compound signal X(n,s) then undergoes three major steps: (1) robust estimation of stride times by employing component-wise and fused estimation; (2) an assessment of stride positions; (3) extraction of gait sequences; and (4) fusion and enhancement of assessed stride positions. A schematic representation of the proposed procedure is depicted in Figure 1.

### 2.1. Robust Estimation of Stride Times

The estimation procedure is performed by an iterative approach that at *k*-th time instant estimates the current stride time. This is performed by employing analysis windows defined as $W_{k}\left(\nu ,s\right)=\left[w_{k,s_{1}}\left(\nu \right)\ldots w_{k,s_{L}}\left(\nu \right)\right]$ denoted by component-wise analysis windows $w_{k,s}\left(\nu \right)=X\left(n_{k}+\nu ,s\right),\;\nu \in \left\{-N_{\max},\ldots ,N_{\max}\right\}$, where $n_{k}$ stands for the central position of *k*-th analysis window that increases according to predefined step η, and ν stands for lag in a manner that the analysis window takes into account at least two consecutive strides and corresponds to the maximum admissible interval length $N_{\max}$.

#### 2.1.1. Component-Wise Estimation of Stride Times

In the first phase, stride times for all *k*-th analysis window positions are estimated for each component *s*. Sets of times between two strides $N_{k,s}$ are computed by the local cyclicity estimation function F(.) applied to each analysis window that corresponds to the *k*-th time instant applied as a probabilistic Bayesian approach, as proposed in [31]:

(2) $$\forall k,s:N_{k,s}=F_{s}\left(w_{k,s}\right)=\underset{N}{\arg\max}\;p\left(N|\theta_{1}^{s}\right)\ldots p\left(N|\theta_{e_{L}}^{s}\right),\;N\in \left\{N_{\min},\ldots ,N_{\max}\right\}$$

where $\theta_{1},\ldots ,\theta_{e_{L}}$ stand for the set of preselected combinations of local cyclicity estimators, $N\in \{N_{\min},\ldots ,N_{\max}\}$ stands for the interval of discrete admissible stride times, and p(N|θ) represents posterior probability density function. In this manner, the most likely joint estimate of stride time is obtained for the *k*-th analysis window. The maximum probability that determines the most likely stride time also serves as a confidence value $v_{k,s}$ for the *k*-th analysis window in the *s*-th component obtained as $v_{k,s}=\max_{N}\;p\left(N|\theta_{1}^{s}\right)\ldots p\left(N|\theta_{e_{L}}^{s}\right)$. Values $v_{k,s}$ are crucial in the fusion process of stride positions and reflect the importance of the currently estimated *k*-th stride time within each component *s*.

#### 2.1.2. Fused Estimation of Stride Times

Stride times are estimated as a joint probability by combining all preselected cyclicity estimators in all sensing modalities in order to determine the most likely *N* from $\left\{N_{\min},\ldots ,N_{\max}\right\}$ implicitly in a global context:

(3) $$\forall k:N_{k}=F\left(W_{k}\right)=\underset{N}{\arg\max}\;p\left(N|\theta_{e_{1}}^{s_{1}}\right)\ldots p\left(N|\theta_{e_{L}}^{s_{1}}\right)p\left(N|\theta_{e_{1}}^{s_{2}}\right)\ldots p\left(N|\theta_{e_{1}}^{s_{L}}\right)\ldots p\left(N|\theta_{e_{L}}^{s_{L}}\right)$$

Such a length estimation procedure is used for a robust assessment of stride times in a global sense, and is applied for gait sequence extraction, enhancement of detected stride positions, and final reconstruction. For this purpose, we also introduce function g(n) that for arbitrary position *n* where n≤ℓ with resolution η determines the corresponding stride time for all values $N_{k}$ obtained by nearest-neighbour interpolation.

#### 2.1.3. Local Cyclicity Estimators

We introduced local cyclicity estimators θ so that for each interval *N* from the set of admissible stride times $\left\{N_{\min},\ldots ,N_{\max}\right\}$ we determined local cyclicity in terms of stride time probability for each position of the analysis window. Several either simple or advanced estimators of different types can be applied. The basic idea behind using local cyclicity estimators is that each of them plays its own role within the joint probability estimation procedure. As described in [31], for instance, correlation-based methods are robust against noise and have different noise characteristics. Conversely, shape-similarity-based methods can be also applied in a context of similarity estimation of two adjacent observations (inter-stride similarity). In such a case, similarity-based methods like dynamic time warping (DTW) exploit and compare the gait morphologies of two consecutive strides. Different characteristics of estimators are exploited in a manner that they complement each other upon fusion. Thus, the following four estimators that have proven their value by physiological parameter estimation (e.g., heartbeat, gait, etc.) were examined: (1) autocorrelation (Rabiner et al. [33,34], Brüser et al. [31]); (2) average magnitude difference function (Ross et al. [35], Brüser et al. [31]); (3) maximum amplitude pairs (Brüser et al. [31]); and (4) dynamic time warping (DTW) (Barth et al. [10]).

### 2.2. Assessment of Stride Positions

Fiducial points are defined as pairs $\left\{\mathrm{position},\mathrm{amplitude}\right\}$ that indicate a particular action or event in a single stride (heel strike, mid-stance, toe off, etc.). The properties of fiducial points depend on many factors, including sensor instalment (i.e., fixed in shoes, carrying in pocket, etc.), body location (i.e., hip, foot, etc.), and a particular component or channel (i.e., acceleration on the *x* axis, acceleration magnitude, etc.). Furthermore, in the environment where sensor instalment and body location are not known in advance (i.e., smartphone in pocket), such points do exist but usually cannot be strictly determined and directly associated with specific gait events [12]. For example, prominent local extrema within an acceleration magnitude signal obtained during naturalistic walking can reflect abrupt changes that may indicate a particular gait event (i.e., heel strike).

The aforementioned properties can be exploited by constructing a procedure for assessment of fiducial points. We begin using estimated stride times between two consecutive strides $N_{k,s}$ that in fact represent a temporal distance between two fiducial points. Exactly two fiducial points are expected to appear in a particular analysis window, one in the left part and another in the right part of the window for the *k*-th analysis window of the *s*-th component. Let $M_{k}$ represent a set of fiducial point candidates in the right half of the *k*-th analysis window *w*. The global position of the proper fiducial point can be then extracted by using function f(.) defined with regards to the type of observed fiducial points in the context of the observed gait event within the *s*-th component:

(4) $$t_{k,s}=n_{k}+f\left(w_{k,s}\left[m\right],N_{k,s}\right),\;m\in M_{k}\wedge n_{k}\leq m\leq N_{k,s}$$

Functions that define fiducial points can be either general or specific. From the perspective of applicability, they can be employed automatically or defined by the user in advance. Let us state some typical examples. The first example is heel strike as a gait event observed by inertial sensors embedded in shoes, where the inertial signal abruptly decelerates when the heel hits the surface [18]. This is reflected as local extrema (either maxima or minima) within inertial signals. In this case, f(.) should be defined as $f_{\mathrm{MAX}}\left(x_{1}\left[m\right],D\right)=\underset{m}{\arg\max}\left(x_{1}\left[m\right]+x_{1}\left[m-D\right]\right)$ or $f_{\mathrm{MIN}}\left(x_{1}\left[m\right],D\right)=\underset{m}{\arg\min}\left(x_{1}\left[m\right]+x_{1}\left[m-D\right]\right)$, respectively. Another interesting example for the definition of fiducial points in terms of gait analysis are zero-crossings. As described in [18], fiducial points represented as a zero-crossing can be applied for detection of the toe-off of the gait by employing inertial sensors embedded in shoes that indicate the gait event where the ankle joint changes its position from a flexion to an extension. In such cases, fiducial points based on zero-crossings can be detected by employing function $f_{\mathrm{ZC}}\left(x_{1}\left[m\right],D\right)=\underset{m}{\arg\min}\left|\left|x_{1}\left[m\right]\right|-\left|x_{1}\left[m-D\right]\right|\right|$. In the same manner, function f(.) can be defined in similar way for other types of fiducial points (i.e., points of inflection, peak prominence, zero energy, etc.).

In contrast, it is known that in the case of signals acquired in uncontrollable scenarios one cannot determine fiducial points explicitly in a context of gait events. However, it is certain that fiducial points are always manifested within inertial signals in a discriminative way due to the cyclostationary property of gait and admissible complexity of gait cycles. It turns out that by sensors attached on body locations with sufficient motion dynamics, complex gait patterns and fiducial points can be assessed automatically in terms of prominent local extrema.

#### 2.2.1. Stride Position Assessment Procedure

Since step η is intentionally considerably smaller with respect to the length of analysis window, multiple replicated values $t_{k,s}$ are obtained for a particular fiducial point as it covered by many analysis windows. A set of interval-length candidates $D_{i}$ for the *i*-th interval that corresponds to the *i*-th unique fiducial point is obtained as $D_{i}=\left\{d_{k,s}|t_{k,s}={\hat{t}}_{i,s}\right\}$. The estimated interval length ${\hat{d}}_{i,s}$ is then obtained as:

(5) $${\hat{d}}_{i,s}=D_{i}^{U}\left(j\right),\;j=\arg\max R\left(D_{i}^{U},D_{i}\right)$$

where $D_{i}^{U}$ stands for a set of unique values of set $D_{i}$ and R(A,B) represents an operation that determines the number of appearances for each element from the set A within a set B. Furthermore, the confidence measure of *i*-th fiducial point for the *s*-th component is determined as a mean value of the corresponding values for unique *i*-th fiducial points:

(6) $${\hat{v}}_{i,s}=\frac{1}{P({\hat{t}}_{i,s})}\sum_{\left\{k|t_{k,s}={\hat{t}}_{i,s}\right\}}v_{k,s}$$

where P(.) stands for the number of elements in set. Thus, for each component, a set of triplets $\left({\hat{d}}_{i,s},{\hat{t}}_{i,s},{\hat{v}}_{i,s}\right)$ respectively representing the estimated interval length, time position, and confidence measure of a particular stride is obtained. As a consequence of noise (i.e., high-frequency oscillations) that can appear in inertial/magnetic signals, misplaced detections in a range of few samples around proper locations may appear when employing f(.) on each analysis windows. To address this issue, we start from the fact that due to step η, the number of expected unique replicated stride estimations that appear around the *i*-th position is known in advance and is denoted as $l_{\exp}$. Consequentially, the number of detected stride estimations denoted by $l_{\mathrm{cnt}}$ should be close to the expected number of stride estimations. Thus, the following triplets are only preserved by setting the value *h* close to 1:

(7) $$\left\{\left({\hat{d}}_{i,s},{\hat{t}}_{i,s},{\hat{v}}_{i,s}\right)|\frac{l_{\mathrm{cnt}}\left(i\right)}{l_{\exp}\left(i\right)}>h\right\}$$

where the number of expected replicated estimates is computed as

(8) $$l_{\mathrm{cnt}}\left(i\right)=\left\{P\left(t_{k,s}\right)|t_{k,s}={\hat{t}}_{i,s}\right\}\;\mathrm{and}\;l_{\exp}\left(i\right)=\frac{{\hat{t}}_{i,s}}{\eta }.$$

#### 2.2.2. Removal of Unreliable and Incorrect Strides

The obtained set of triplets may also contain strides that may reflect low stride confidence through stride confidence value $v_{i}$. Besides that, it can also contain incorrect strides detected due to sensor variability noise in the phases of inactivity or periodic motion noise. To remove those unreliable and incorrect detections, two types of fixed and robust thresholds can be applied: stride confidence threshold $h_{v}$ and noise variability threshold $h_{r}$. Threshold $h_{v}$ is defined directly with stride confidence value $v_{i}$—all strides with value $v_{i}$ lower than $h_{v}$ are omitted from the set of triplets. Threshold $h_{r}$ can be directly defined with the dynamic range of the observed phenomena for a particular sensor component, i.e., either on the measured variability of sensor noise or minimal expected dynamic range if the activity level of the observed phenomena is known in advance (i.e., gait).

The procedure of setting these thresholds does not assume much effort. In fact, their use is optional and strictly depends on the nature of observed data. It turns out that a single fixed value for a particular threshold, either for a specific sensor component or a combination of estimators, is enough. Further insight and practical details are given in Section 2.5.

### 2.3. Extraction of Gait Sequences

Gait sequences are extracted with a special backtracing procedure. It assumes that the assessed strides represented by triplets $\left(d_{i},t_{i},v_{i}\right)$ are sorted in time. Then, each estimated stride time $d_{i}$ on position $t_{i}$ is compared to the corresponding globally estimated one through g(i) at the same time instant. It is expected that times between detected strides $d_{i}$ will be close to those estimated with $g(t_{i})$. Therefore, we rely on the assumption that the absolute difference between these values defined as Δ will stay within $N_{m}in$, as the values larger than $N_{m}in$ will appear only in situations where strides are not detected—either because the user stopped walking (sequences with silence) or as a result of other external disturbances that affect gait pattern (i.e., activity noise).

The procedure starts with the latest assessed stride position, which is determined as end of the last gait sequence. If value Δ obtained by comparing it with the previous stride position is smaller than $N_{m}in$, then the previous stride is added to the current gait sequence. If not, the initial stride position (onset) of the current gait sequence is determined by employing value $d_{i}$ of the current stride and the corresponding definition function for fiducial points f(.). The previous stride then represents a final stride (termination) of a new gait sequence. The procedure completes its work when all triplets are addressed. Consequentially, gaps between sequences represent phases with silences. The output of the procedure is represented by the set of onsets and terminations for each component in the form of pairs $\left(S_{\mathrm{onset}}\left(j\right),S_{\mathrm{end}}\left(j\right)\right)$ for the *j*-th sequence.

### 2.4. Fusion and Enhancement of Stride Positions

The fusion and enhancement procedure of stride positions is applicable in cases when selection of the reference component and extraction of the most reliable fiducial points is automatized. This is an efficient solution for addressing the problem of transitions and dynamically changing gait patterns acquired by measuring gait in uncontrollable scenarios. We start from stride positions and corresponding gait sequences obtained in the previous step. First, for each *j*-th sequence, a reliability score is estimated as the mean absolute error between $d_{i}$ and $g(t_{i})$ corresponding to the strides within each sequence. In descending order according to the assessed mean absolute error, sequences are added to the global output. Sequences that overlap each other are merged together. The procedure is finished when the entire temporal space is covered.

A single reference component can be selected either manually or automatically. Manual selection of the reference component can be also applied in the case when the most reliable component is identified in advance, i.e., as the gyro *z*-axis in shoe sensors for determining zero velocity [18]. In case of automatic selection, the best single-reference component $s_{\mathrm{ref}}$ is determined as:

(9) $$s_{\mathrm{ref}}=\underset{s}{\arg\min}\left\{\frac{1}{P(\Delta t_{s})}\sum_{i}\left|g\left({\hat{t}}_{i,s}\right)-\Delta {\hat{t}}_{i,s}\right|\right\},\;\Delta t_{i,s}=\left\{{\hat{t}}_{i,s}-{\hat{t}}_{i-1,s}\right\},\;i>1$$

In the subsequent step, initial temporal alignment of the particular strides on the reference component $s_{\mathrm{ref}}$ per *j*-th sequence is performed for all components *s*. Let ${\hat{r}}_{s}\left(n\right)$ represent the compound sparse vector obtained as the linear combination of Kronecker delta function on the estimated stride time instants with stride confidence values $v_{i,s}$:

(10) $${\hat{r}}_{s}\left(n\right)=\sum_{i}{\hat{v}}_{i,s}\delta \left(n-{\hat{t}}_{i,s}\right).$$

As stride confidence values $v_{i,s}$ stress the importance of *i*-th stride in the context of a particular component *s*, strides with higher confidence will be reflected as pulses with higher values in sparse vector ${\hat{r}}_{s}\left(n\right)$. Thus, time instants are aligned according to fixed lags $\ell_{s}$ obtained by normalized cross-correlation between reference sparse vector ${\hat{r}}_{s_{\mathrm{ref}}}$ and all remaining sparse signals for the *j*-th sequence:

(11) $$\forall s,s\neq s_{\mathrm{ref}}:\;\ell_{s}=\underset{\tau }{\arg\max}\left|\left({\hat{r}}_{s_{\mathrm{ref}}}⋆{\hat{r}}_{s}\right)\left(\tau \right)\right|,\;\tau \in \left\{-N_{\max}\ldots N_{\max}\right\}$$

where lag τ is constrained according to physiological boundaries of the gait frequency. Aligned sparse vectors are then obtained as lagged versions of vectors ${\hat{r}}_{s}$ by lags $\ell_{s}$ for the particular component *s* on each of the *j* gait sequences:

(12) $$r_{s}\left(n\right)={\hat{r}}_{s}\left(n-\ell_{s}\right)$$

It is expected that this will significantly reduce the effect of delay specific for different types of fiducial points.

The fusion procedure is initiated by construction of compound sparse matrix ${\tilde{Y}}_{s}\left(m,n\right)$, obtained as:

(13) $$\tilde{Y}\left(m,n\right)=\left[\begin{array}{ccc} r_{s_{1}}\left(n\right) & \ldots & r_{s_{L}}\left(n\right) \end{array}\right]$$

Non-zero values representing the confidence measure of detected strides will appear column-wise. Thus, the marginal projection of sparse matrix $\tilde{Y}\left(m,n\right)$ over a temporal dimension is computed as $\tilde{y}\left(n\right)=\sum_{m=1}^{P(s)}\tilde{Y}\left(m,n\right)$. The exact positions of fiducial points that represent stride positions are obtained by window-based fusion, performed by convolution of marginal projection $\tilde{y}\left(n\right)$ using triangular window h(n) as $y\left(n\right)=\left(\tilde{y}*h\right)\left(n\right)$ with length *L* set to $N_{\max}$. In this manner, overlaying confidence values are emphasized while outliers are suppressed. Peak values are then gathered in a set that can be seen as a simple two-class problem. Then, reliable strides are simply extracted by an automatically determined threshold obtained by applying a kernel density estimation of the distribution, and the others are omitted.

To finalize the enhancement procedure, in order to remove possible slight displacements from fiducial points defined by the reference component, let the pairs $\left(t_{i}^{\max},y_{i}^{\max}\right)$ represent all detected maxima peaks and their positions and $\left(t_{i}^{\min},y_{i}^{\min}\right)$ represent minima peaks adjacent to $\left(t_{i}^{\max},y_{i}^{\max}\right)$ computed from y(n). Final strides are then assessed as triples $\left(d_{i},t_{i},v_{i}\right)$, where $d_{i}=t_{i+1}^{\max}-t_{i}^{\min}$, $t_{i}=t_{i}^{\max}$ and $v_{i}=\frac{1}{2}\left(\left|y_{i}^{\max}-y_{i}^{\min}\right|+\left|y_{i}^{\max}-y_{i+1}^{\min}\right|\right)$.

### 2.5. Evaluation Parameters

Gait sequences are defined as time intervals that cover consecutive strides corresponding to a single comprehensive gait trial. The performance of gait sequence extraction was carried out in terms of gait sequence coverage on the basis of individual time samples. Each time sample corresponds to one of the following four classes: (1) true positive (TP): a time sample that corresponds to the gait sequence in both ground truth and observed data; (2) true negative (TN): a time sample that does not correspond to the gait sequence in both ground truth and observed data; (3) false positive (FP): a time sample that does not correspond to the gait sequence in ground truth data but was falsely detected as part of the gait sequence in observed data; and (4) false negative (FN): a time sample that corresponds to the gait sequence in ground truth data but was pronounced as undetected in the observed data. Based on these four classes, we provide standardized metrics of sensitivity TP/(TP+FN), specificity TN/(FP+TN), precision TP/(TP+FP), recall FP/(FP+TN), and F-score, computed as $2\cdot \frac{precision\cdot recall}{precision+recall}$.

By evaluating stride segmentation performance, detected strides were divided into three groups. The detected stride is declared as a TP if its time instant falls within ±100 ms of the labelled stride time borders as suggested in [10]. All other detected strides that fall within labelled stride borders (except for the TP which is in this case the closest to the manually labelled stride time instant), are declared as FPs. This also includes strides that were detected outside manually segmented regions. Finally, if no stride is detected in a particular labelled ground truth stride time border, the missing stride is declared as an FN. Based on these three classes, the performance of a proposed stride detection procedure is evaluated with precision, recall, and F-score.

Since the proposed approach assumes a single stride confidence threshold from interval [0,1] that determines whether a particular stride should be considered or not, its value should be determined prior to the detection procedure. We followed the concept proposed in [10], where the best threshold for each combination of a particular population and used sensor axes was selected. Thus, a single stride confidence threshold is determined for each of 30 combinations of the considered evaluators and components. To prove the stability of the stride confidence threshold, precision and recall curves were computed for each trial by varying the threshold with values that correspond to a proper co-domain. The threshold at which the intersection of both curves appear is considered as the optimal threshold that reflects the best trade-off between FP and FN strides. The final stride confidence threshold for the observed method is then obtained as a mean value of these optimal thresholds. This approach to threshold selection is justified by the assumption that the variability of threshold values around the mean threshold will be low in the case of a stable and reliable recognition system. Therefore, standard deviations of values around selected stride confidence threshold values are also observed in parallel.

Concerning stride positioning accuracy, one of of the most common used evaluation approaches is to observe detected positions through the time intervals between two consecutive stride positions. Therefore, let $d_{i}$ represent the estimated stride time for the *i*-th time position and $d_{i}^{\mathrm{ref}}$ its corresponding ground truth stride time. Absolute errors between estimated and ground truth stride times can be then computed as $AE=|d_{i}-d_{i}^{\mathrm{ref}}|$. From the set of estimated absolute errors, the following four metrics are computed: mean absolute error, median absolute error, dispersion of errors by standard deviation of absolute errors, and $P_{95}$ of absolute errors yielding the value with 95% of errors below it.

Furthermore, the agreement between estimated and ground truth stride times is shown by regression analysis, performed by employing least-squares regression on ground truth and corresponding detected stride times, and Bland–Altman plots that depict the differences between corresponding estimated and ground truth stride times ($d_{i}^{\mathrm{ref}}-d_{i}$) against the mean value of stride times ($\frac{d_{i}^{\mathrm{ref}}+d_{i}}{2}$). Stride positioning bias is another important aspect that should be observed during the evaluation procedure. Bias can be assessed in terms of reference-to-detected delay of fiducial points. A set of delays for all strides can be thus computed as the difference $t_{i}-t_{i}^{\mathrm{ref}}$. A positive value reveals the number of samples for which the fiducial point was detected after an actual fiducial point, and vice versa. Furthermore, low dispersion of these delays additionally confirms the accuracy of fiducial point positioning and can be expressed by the standard deviation of the delays.

## 3. Experimental Datasets

### 3.1. FRIgait Dataset

We have performed a continuous collection of inertial/magnetic data in order to construct dynamically growing, large-scale gait dataset collected from a large number of users by employing their smart devices in the course of their daily lives. The FRIgait dataset is planned to be used as a reference for the evaluation of gait assessment procedures during daily life since it provides comprehensive coverage of gait-affecting parameters (footwear, health state, surface, etc.), the long-term monitoring ability of a subject’s gait profile, and reduced awareness of the subject that he/she is being monitored. The dataset is collected by our own developed cloud-based IoT integration platform that enables simple connectivity of smart devices and continuous collection of inertial gait data by a large number of users. Widespread gait monitoring is simplified by an intuitive mobile application that also gathers user-based gait-related auxiliary information for a single walking trial. This information is used for evaluation purposes after data analysis and includes data on gait-affecting patterns, walking manner, health state, etc.

**Figure 2:**
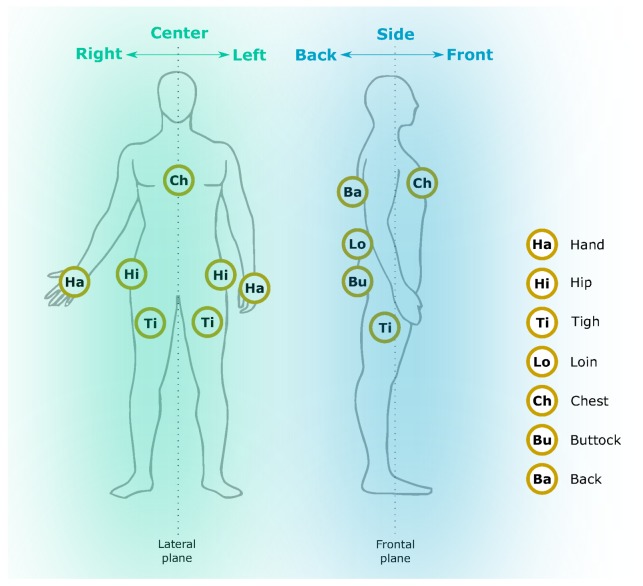
Layout of all smartphone positions considered in the FRIgait dataset.

**Figure 3:**
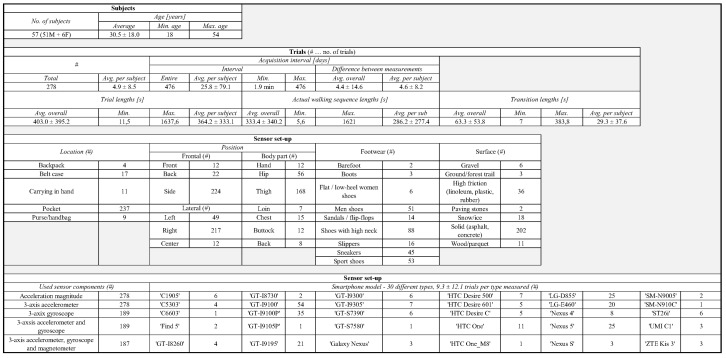
Properties of the FRIgait dataset.

The current version of the FRIgait dataset contains data collected over approximately 1.3 years. Fifty-seven subjects ranging from ages 18 to 54 provided 278 walking trials lasting approximately 5.5 ± 5.7 min, where the longest walking trials lasted up to 30 min. The dataset includes gait data employing different footwear and surfaces. It also includes regions with transitions. The data is measured with more than 30 smartphone types. Besides that, data was collected from multiple body locations according to the lateral and frontal plane—please refer to Figure 2 for the sensor positioning layout. Complete details on the proposed FRIgait dataset are given in Figure 3. In order to evaluate or directly compare the results of gait segmentation or other related gait analysis approaches, authors are encouraged to run their algorithms on the FRIgait dataset. The dataset is open and available upon request.

The FRIgait dataset contains inertial/magnetic data that is very appropriate for reliable evaluation of the proposed gait segmentation approach as it includes sudden changes in gait frequency, abrupt morphological changes in gait patterns, sequences with silences, and other activity sequences (i.e., due to taking or putting the smartphone in measuring position), as well as gait patterns with varying morphology, i.e., gait patterns using a smartphone stored in a purse provides different information than when stored in a pocket.

After acquisition, auxiliary information on gait provided by users through the mobile application was extracted for each trial. Manual extraction of gait sequences and manual labelling of all stride time instants was then performed for each trial by three different persons. In order to reduce manual labelling bias, a majority voting scheme was performed on each stride to determine whether the stride was valid or not. Strides were labelled sequentially according to the auxiliary information and stride prominence induced in inertial/magnetic signals, i.e., strides reflected as the most prominent peaks or the most discriminative repetitive patterns. Thus, each stride label appears in at least one component that represents the most distinguishable repetitive pattern. Besides that, we have also labelled signal segments that determine transition phases.

### 3.2. eGait Dataset

In order to examine whether the proposed approach can be also directly applied in more demanding and special measuring environments, where high stride segmentation accuracy should be achieved, the *eGait* dataset collected at FAU Erlangen-Nürnberg provided by Barth et al. [10] was used in the evaluation procedure. This dataset consists of inertial data acquired by two shoe-mounted inertial sensors (each having a 3-axis accelerometer and gyroscope) that were mounted laterally on the heels of the subject’s right and left shoes. The experiment included three cohorts: geriatric individuals, PD patients, and healthy (control) subjects. Measurements were performed by considering two different protocols. The first involved 4 × 10-m-long controlled walks at a comfortable self-selected walking speed, while the second involved free walking, where subjects walked for two minutes inside a building. This included straight and curved walking, stair climbing, sit-to-stand transitions, standing, opening and closing the doors, etc. For each of three cohorts, 15 subjects participated in the experiment, where 10 subjects performed controlled walking, and five subjects performed free walking. All stride segments were labelled manually by considering angular velocity in the sagittal plane. For other details on measurement, please refer to the original publication [10].

By employing this dataset, we want to assess the general applicability of the proposed approach by its evaluation in specific conditions that differ from the concept covered by our FRIgait dataset. It is expected that fixed positioning of sensing devices should additionally contribute to the improvement of stride detection accuracy. Finally, evaluation results presented in [10] will be directly employed in order to make a direct comparison of results of the proposed approach with peak-detection-approach proposed by [36] and the template-based approach proposed by authors of the eGait dataset [10].

### 3.3. Experimental Parameters

**Figure 4:**
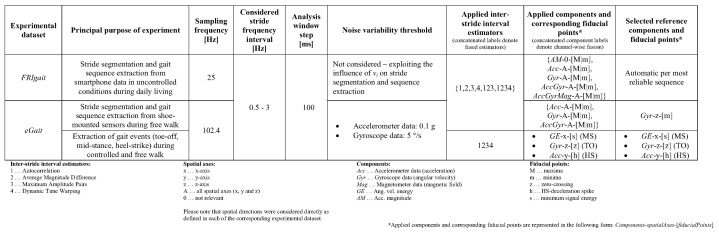
Experimental parameters used for evaluation of the proposed approach on FriGait and eGait datasets.

We examined the performance of the proposed approach using several experimental parameters for both employed datasets. All details on the experimental parameters used the evaluation procedure are shown in Figure 4. For better comprehension, we introduce unified labels for applied local cyclicity estimators, sensor components, and fiducial points. All labels are provided in Figure 4. To present applied combination of cyclicity estimators, components, and fiducial points, the following form is used: *estimator-Components-spatial_axes-[fiducial_points]*. For example, 123-*Acc*-A-[M|m] represents the combination of cyclicity estimators labelled as 1, 2 and 3, with acceleration data on all three spatial axes, and separately applies local maxima and minima as fiducial points. This would result in six output components (all estimators applied on three spatial axes with two types of fiducial points for each axis).

The sampling frequency that was used in the evaluation procedure was equal to 25 Hz for the FRIgait dataset and 102.4 Hz for the eGait dataset. This decision is tightly related to measuring concepts that each of these two dataset originally address. First, for the purpose of efficient measurement with smartphones it is desired that the sampling frequency is as low as possible, i.e., low power consumption. It has been already shown that the sampling frequency 25 Hz is in this situation sufficient for satisfactory resulting processing of gait measured with inertial sensors [3]. An equidistant sampling interval has been achieved by employing linear interpolation on raw collected data that was sampled by varying the highest possible sample rate, which depended on the type of the smartphone. In contrast, the sampling rate by eGait is considerably higher and was left unchanged in order to reduce accuracy error on the sample level (approximately 10 ms compared to 40 ms by sampling frequency of 25 Hz) and to be directly comparable by the approach presented in [10].

Concerning a priori knowledge on gait frequency, according to Oberg [37], the expected human physiological limitations on stride frequency were set to within the interval of between 0.5 Hz and 3 Hz. These values were employed to determine expected values of $N_{\min}$ and $N_{\max}$ that were set to 0.33 s and 2 s, respectively. It should be mentioned that minimum are maximum stride frequencies that appear in both datasets are equal to 0.55 and 1.2 Hz, respectively. Analysis window step η was set to the length of approximately 100 ms, which about 30% of the shortest expected inter-stride interval length. The noise variability threshold $n_{r}$, which was applied on the eGait dataset only, addressed sensor noise variability and was set to 0.1 g for acceleration data and 5 degrees/s for angular velocity.

For the evaluation procedure, we constructed a test framework that contains the following local cyclicity estimators: auto-correlation, inverted average magnitude difference, maximum amplitude pair-based estimators as fundamental frequency-observing estimators, and the DTW-based estimator as a similarity-based estimator. These were labelled with the labels ‘1’, ‘2’, ‘3’, and ‘4’, respectively. We also experimented with two fused versions of estimators, one labelled with ‘123’ as a fusion of all fundamental frequency-observing estimators, and one with the added similarity-based estimator, labelled as ‘1234’. Concerning employed sensor components, we experimented with the following sensor channels or their combinations: acceleration magnitude computed from 3-axial accelerometer measurements (a single component), 3-axial accelerometer measurements (three components), 3-axial gyroscope measurements (three components), angular velocity energy (single component), fusion of 3-axial accelerometer and gyroscope measurements (six components) and fusion of 3-axial accelerometer, gyroscope, and magnetometer measurements (nine components). The proposed approach was performed on datasets with all combinations of pairs *estimator-component* of aforementioned constructs, where each pair can be defined as single method (i.e., ‘1234-*AccGyr*’). In this manner, 30 (6 · 5) methods were constructed for the FRIgait dataset in order to get insight into a possible trade-off between performance and computational complexity in terms of efficiency, since the increased number of applied estimators or components can lead to increased computational complexity of the proposed approach.

To evaluate the accuracy of the positioning of fiducial points, we began with the ground-truth labelled data provided in the FRIgait and eGait datasets. To show the generality of the proposed approach, the evaluation setup for stride segmentation was set the same for both datasets and relied on extraction of fiducial points in terms of local maxima by applying function $f_{\mathrm{MAX}}$ and local minima by $f_{\mathrm{MIN}}$. For example, an estimation trial ’1234-*AccGyr*’ would result in segmented six sensor channels by employing information on estimated stride times obtained by fusing four local cyclicity estimators. As the FRIgait dataset contains free-walk data with varying sensor positions, prominent peaks can be determined as fiducial points, but the morphology of the gait pattern is not expected to be preserved. Since the labelled data in the FRIgait dataset is set on the most prominent peaks and the selection of the best component and peak positioning may vary with time, fiducial points were selected automatically. This also covered transition phases, since it was expected that the proposed approach would preserve robustness on the account of some lost strides due to initialization of the new gait sequence within the transition phase. Conversely, due to the fixed sensor attachment in the eGait dataset, fiducial points can be determined as stable and can be also interpreted as gait events due to regular and stable gait patterns. Therefore, reference fiducial points could be defined in advance. As described by Barth et al. [10], reference stride boundaries were in their dataset obtained by employing angular velocity in the sagittal plane, where prominent negative peaks that determine the change in the foot rotation during one stride were used in order to define stride start and end points (*Gyr*-z-[m]). By evaluating our approach with the eGait dataset, we directly adapted these positions as fiducial points during the stride enhancement step. Finally, in order to examine whether the proposed approach was able to accurately determine gait events without any templates and in unsupervised way, we processed the eGait dataset with the proposed approach by employing the same components and fiducial points as proposed in [18].

## 4. Results

### 4.1. Smartphone Data during Free Walking

**Figure 5:**
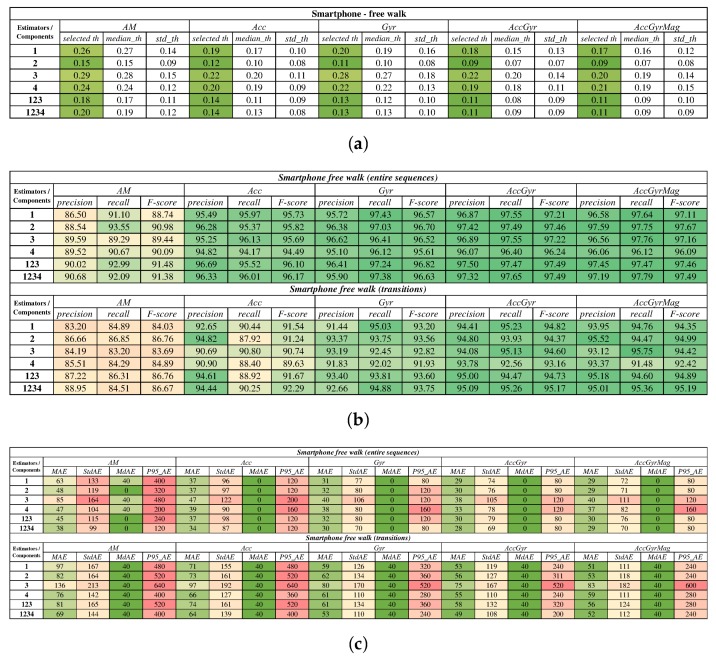
Overall stride segmentation results for smartphone data: (**a**) stride confidence threshold variability analysis based on $v_{i}$; (**b**) stride detection efficiency; (**c**) stride detection accuracy. The values are also represented by a green-yellow-red color scale for a better representation.

Concerning stride detection performance of the proposed approach on smartphone dataset, Figure 5 presents the overall results gathered with the combinations of selected cyclicity estimators (1, 2, 3, 4, 123, 1234) and components (*AM, Acc, Gyr, AccGyr, AccGyrMag*). The results are presented separately for entire gait sequences and for transitions. First, estimated stride probability $v_{i}$ was exploited in order to determine the proper stride confidence threshold for each combination. These are revealed in Figure 5a. Stride detection performance for particular combinations of estimators and components is shown in Figure 5b by precision, recall, and an F-score, for both entire sequences and transitions. Overall stride detection accuracy is presented in Figure 5c with average errors.

**Figure 6:**
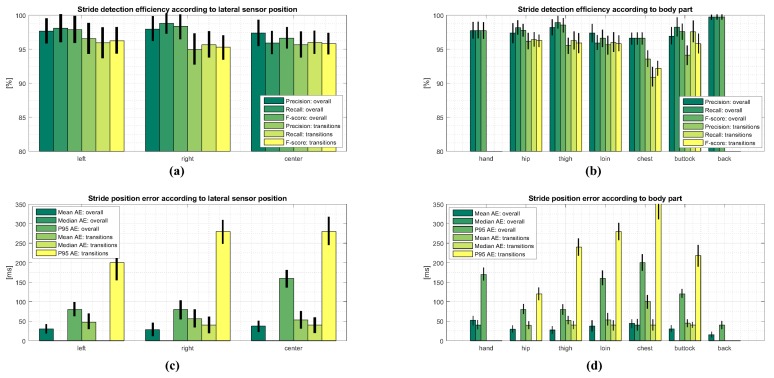
Efficiency and accuracy of stride segmentation according to sensor location. Missing bars represent zero-values. Black bars represent standard deviation. The values are also represented by a green-yellow-red color scale for a better representation.

To examine the influence of sensor body position on stride detection performance, we investigated stride detection efficiency and accuracy according to: (1) lateral sensor position including the left, right and center positions; and (2) the body part on which the sensor was attached during measurements including: hand, hip, thigh, loin, chest, buttock, and back. The obtained results are shown in Figure 6a,b, where precision, recall, and an F-score were computed for overall data and transitions, respectively. Corresponding stride position errors are shown in Figure 6c,d for lateral sensor positions and different body parts, respectively. The figure also includes absolute errors for overall data and transitions. The results of transitions for hand and back data are excluded from the analysis due to an insignificant number of trials covering these particular body parts.

To evaluate stride detection accuracy, we used a combination of stride interval estimators (*1234*) and corresponding components (*AccGyr*-A-[M|m]) that showed the best performance in terms of precision, recall, and F-score. Fiducial points defined with local minima and maxima as an outcome of the proposed approach were extracted automatically as per the most reliable sequence denoted with the highest average $v_{i}$ in extracted sequences. Concerning detection accuracy, first we computed average detection bias as a difference between expected (reference) stride time instants and stride time instants detected by the proposed approach. Overall stride detection bias using a sampling frequency of 25 Hz yielded 0.0372 ± 1.0812 samples, while stride detection bias for transitions resulted in 0.0461 ± 1.4799 samples.

**Figure 7:**
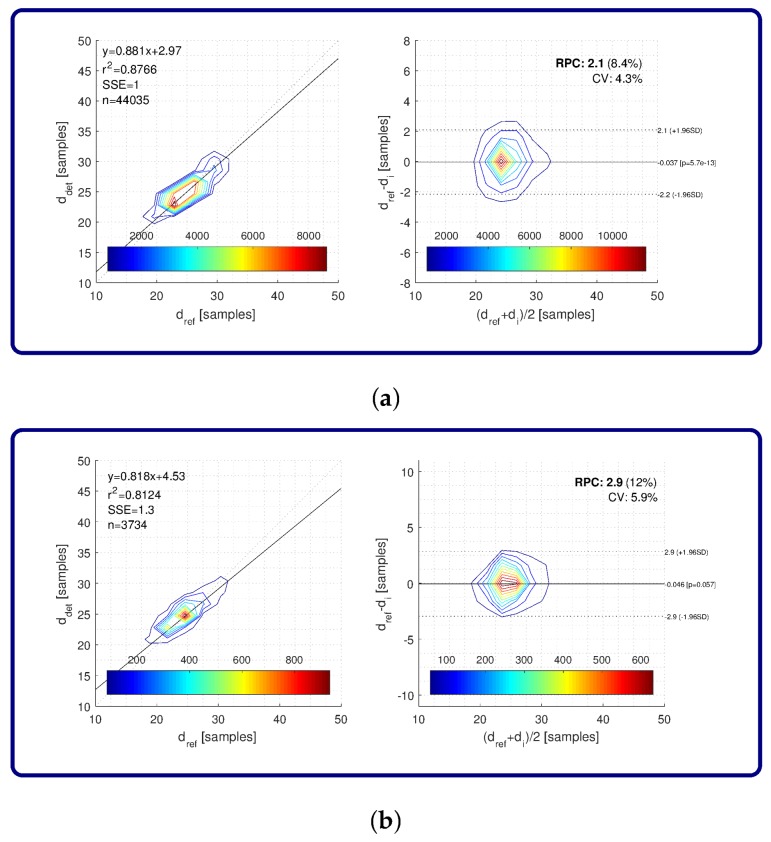
Regression analysis and Bland–Altman plots of stride time matching between detected strides and ground truth: (**a**) all strides; (**b**) transitions. Color map represents the number of strides.

Due to the large number of stride times used in the analysis (*n* = 44,035), matching between detected and corresponding ground truth stride times is visualized by contours that reveal the density of stride times along an estimated regression line (Figure 7a, left-hand side). The same is applied for all regression analysis and Bland–Altman plots in this paper. Regression analysis for all stride times on smartphone data resulted in the coefficient of determination $r^{2}$, with a value of 0.8766 and sum of squared error SSE equal to one sample. Bland–Altman plots for all stride times on smartphone data are depicted in Figure 7a, on the left-hand side, and reveal average values between reference and detected stride times on the *x*-axis and corresponding differences between them on the *y*-axis. The average value of differences between reference and detected stride times yielded 0.037 samples (straight line in the middle of the plot). The reproducibility coefficient RPC was also computed as (±)1.96σ, where σ stands for standard deviation, marking the values that lie within 95% of the differences. In this case, RPC resulted in 2.1 samples. Relative RPC and coefficient of variation CV were also computed and were equal to 8.4% and 4.3%, respectively. We also present the results of regression analysis and Bland–Altman plot for stride times that corresponded to transitions. Results are shown in Figure 7b. In this analysis, 3734 stride times were considered. Concerning regression analysis, the coefficient of determination $r^{2}$ was equal to 0.8124 and SSE was equal to 1.3 samples. The average value of differences was equal to 0.046 samples, RPC resulted in 2.9 samples, and relative RPC and CV were 12% and 5.9%, respectively.

### 4.2. Shoe-Mounted Sensors-Stride Detection

**Figure 8:**
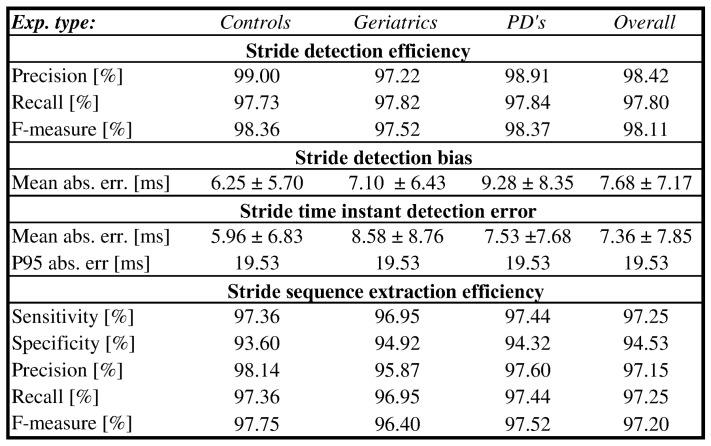
Overall results of the performance of proposed approach on eGait dataset.

Stride detection results for shoe-mounted IMU sensors performed on the eGait dataset [10] are shown in Figure 8, separately for each of the three cohorts, as well as with the overall results. When employing the proposed approach on the eGait dataset, stride time estimation was performed by combining all defined estimators (*1234*) with all available components in all spatial directions (*AccGyr*-A-[M|m]). To be fully compliant with [10], *Gyr*-z-[m] was selected as the reference component when locating stride time instants. Stride detection efficiency as evaluated by precision, recall, and F-measure can be directly compared to the results published in [10]. Figure 8 also reveals the obtained detection bias which seems to stay within one sample. Stride detection accuracy is presented by the absolute value error that falls below one sample and $P_{95}$ below two samples. Results of stride sequence extraction are also presented. At this point it is also important to mention that during the free walking experiment, authors in [10] were interested only in straight walking epochs and considered other walking types (i.e., stair climbing) as false. In contrast with that, we are interested in aggravated circumstances for stride segmentation. Since such circumstances were already covered by the FRIgait dataset and due to the fact that corresponding epochs in eGait dataset are not labelled, these parts were excluded from the analysis in order to ensure fair comparison.

**Figure 9:**
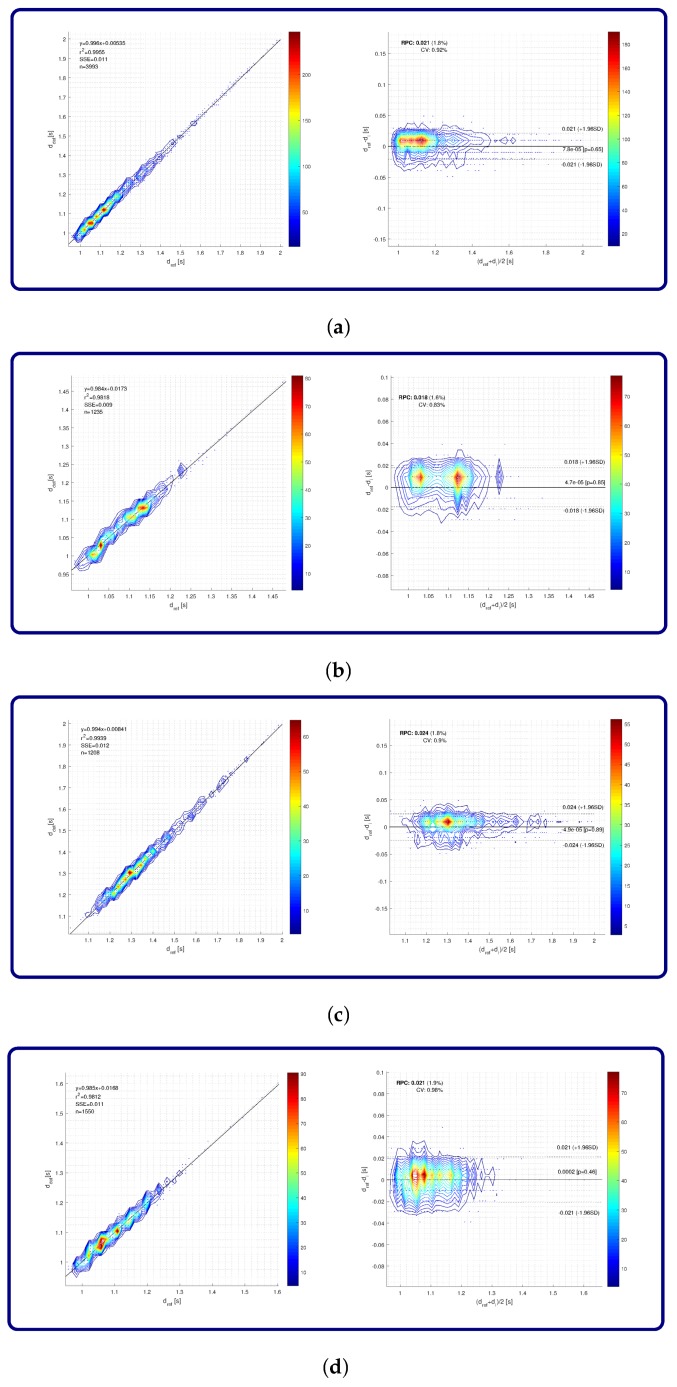
Regression analysis and Bland–Altman plots for stride detection accuracy on the eGait dataset over different cohorts: (**a**) overall; (**b**) control subjects; (**c**) geriatric subjects; (**d**) PD patients. Colour map represents the number of strides.

Regression analysis and Bland–Altman plots for the eGait dataset are presented separately for the overall results and each of the three cohorts. All results are presented in seconds. Figure 9a shows the regression plot and Bland–Altman plot for the overall results. The analysis was performed on 3993 strides. Regression analysis yielded a coefficient of determination $r^{2}$ equal to 0.9955 and SSE of 11 ms. Differences between reference and detected stride times were practically zero on average with RPC value of 21 ms, with relative RPC and CV equal to 1.8% and 0.92%, respectively. The corresponding results for control as well as geriatric subjects and PD patients are depicted in Figure 9b–d, respectively.

**Figure 10:**
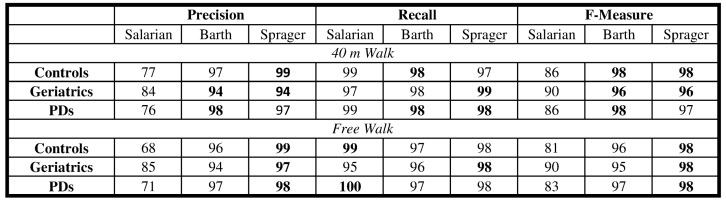
Comparison of the performance of proposed approach with relevant state-of-the-art approaches performed using the eGait dataset.

Figure 10 provides a comparison of stride detection performance with the two most relevant approaches: peak-based detection [36] and the DTW-based approach [10]. The precision, recall, and F-measure are compared separately for each of the cohorts. It should be explained that the results provided by Barth et al. for particular combinations of subject groups and metrics in the figure contain the best results they obtained depending on the combination of components (sensor types) they used. In contrast, our results were obtained by using only one predefined combination of estimators *1234* on all available components in all spatial directions (*AccGyr*-A-[M|m]) and with *Gyr*-z-[m] selected as a reference component, thus exposing the proposed approach as a more universal solution.

### 4.3. Shoe-Mounted Sensors-Detection of Gait Events

**Figure 11:**
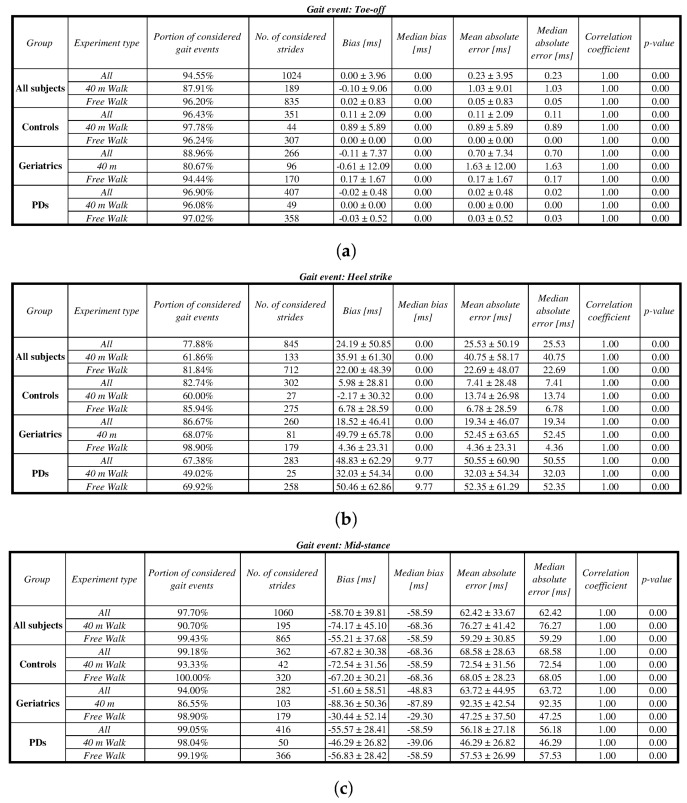
Performance of the proposed approach for detection of gait events, performed on the eGait dataset and validated with eGait system: (a) toe-off; (b) heel-strike; and (c) mid-stance.

In evaluating the performance of gait event detection, ground truth data was determined by the eGait toolbox [18], a verified and reliable framework for assessment of gait parameters. Good matching of detected gait events with those assessed from the eGait toolbox would confirm the proposed approach for the gait event-detection task. We experimented with three types of gait events: toe-off, heel strike, and mid-stance. Obtained results were then directly compared with the corresponding ones from the eGait toolbox which served as ground truth. For stride time estimation, we combined all available estimators (*1234*). Concerning the components and fiducial points, we proceeded in the same way as proposed in [18]. For toe-off detection, we employed the gyroscope *z*-axis with zero-crossings as defined fiducial points (*Gyr*-z-[z]). Heel strike events were determined by employing a function that detects abrupt changes in acceleration on the *y*-axis (*Acc*-y-[h]). Mid-stances were determined by considering the zero-energy of gyroscope data (*GE*-x-[s]). The performance of the proposed approach was evaluated for all data and separately for each of the three cohorts. For each of these, we also separately considered the controlled 40-m walk and free walk. The results of the detection performance are shown in Figure 11a–c for toe-off, heel strike, and mid-stance events, respectively, where further insight into detection performance is given with the evaluation parameters as follows. A portion of considered gait events (coverage) determines the portion of gait events in terms of the ratio between true positives and all reference events. Please note that due to the nature of our approach and strictness of the definition function, there were no false positives detected. We also provide an exact number of strides, considered in an analysis, as well as detection bias (mean, std, and median) against reference gait events. Detection accuracy is estimated by mean, std, and median absolute error between detected and reference gait events. The correlation coefficient and its *p*-value are also computed. When observing the results, one should have in mind that the sampling frequency of 102.4 Hz was used in the analysis, meaning that a one-sample difference corresponds to a time interval of around 9.7 ms.

**Figure 12:**
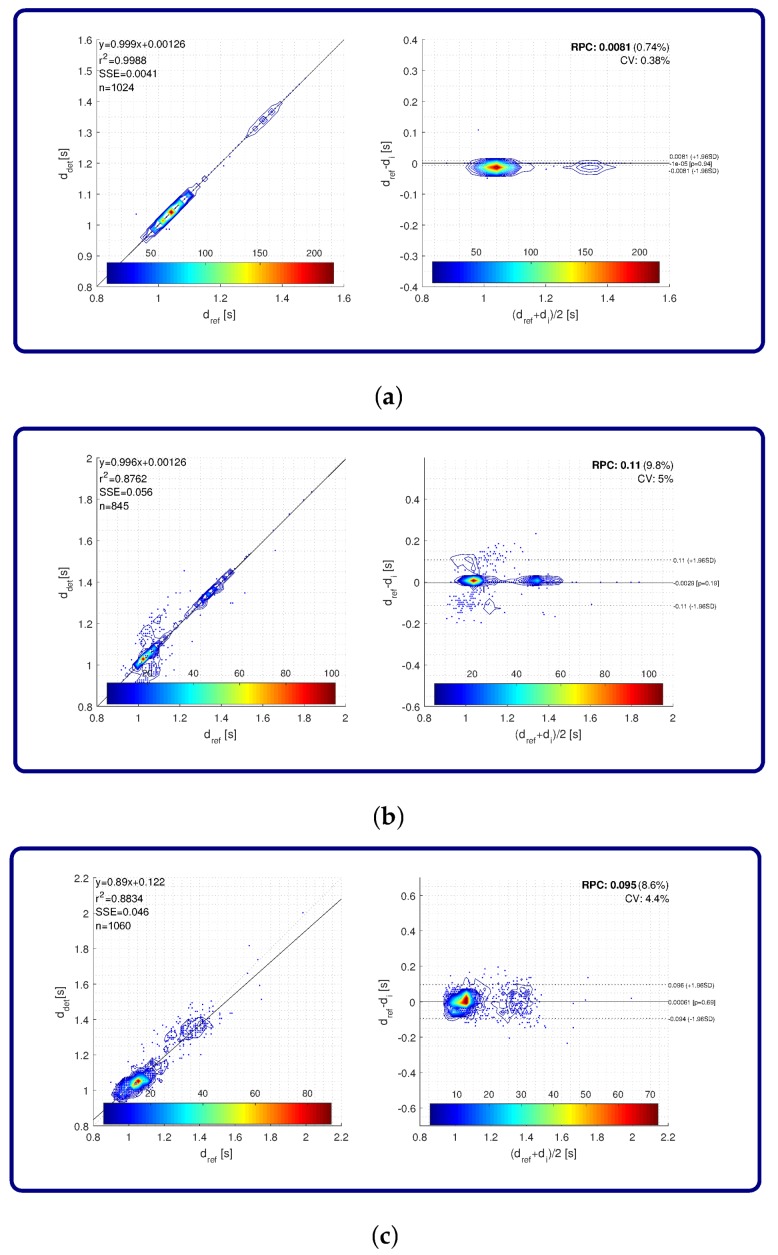
Regression analysis and Bland–Altman plots for accuracy on gait event extraction from the eGait data: (**a**) toe-off; (**b**) heel-strike; and (**c**) mid-stance. Colour map represents the number of strides.

Matching between gait events detected with the proposed approach and ground truth gait events from the eGait toolbox is also evaluated by regression analysis and Bland–Altman plots. A graphical representation of the results is shown in Figure 12. Figure 12a reveals results for toe-off detection. Regression analysis yielded an $r^{2}$ of 0.9988 and an SSE of 4 ms. The difference between reference and detected gait events was practically zero on average with RPC also being very close to zero. Relative RPC and CV were equal to 0.74% and 0.38%, respectively. Concerning heel strike gait events, the $r^{2}$ was 0.8762 and SSE was 56 ms. The difference between the reference and detected gait events was on average also practically zero, while the RPC was 0.11 s, with relative RPC and CV being 9.8% and 5%, respectively. Detected mid-stances resulted in $r^{2}$ equal to 0.8834 and SSE of 46 ms. The difference between reference and detected gait events was on average also practically zero, while the RPC was 0.095 s, with the relative RPC and CV being 8.6% and 4.4%, respectively.

## 5. Discussion

From Figure 5a it can be seen that the stride confidence threshold is stable over all combinations of local cyclicty estimators and components as the standard deviations for each combination are considerably low. Selected values and medians of thresholds are rather stable and converge towards 0.11. That indicates the satisfactory robustness of the proposed approach and good performance also without considering noise threshold. Concerning stride detection performance on the FRIgait dataset, it can be seen from Figure 5b that combining more estimators and more components leads to better results. From these results it is also obvious that magnetic data operating with minima and maxima as gait events does not contribute to the overall efficiency, which is meaningful due to the nature of raw magnetic signals during gait. In general, precision, recall, and F-score are completely comparable with recent state-of-the-art methods regardless of the fact that FRIgait dataset was acquired in more challenging conditions. Evaluation on isolated transitions shows just a 2–3% lower performance when compared to overall results, which is still acceptable and reveals that proposed approach copes well also with more demanding situations. The results of stride segmentation accuracy for the FRIgait dataset are in line with the efficiency—the mean average error for successful combinations of estimators and components is less than one sample. The accuracy (Figure 5c) is slightly poorer for transitions, which was expected due to higher dispersion of detections. Still, 95% of errors fall within the interval of ±0.2 s.

The results also show that stride segmentation performance is not significantly affected by using a measuring device on different body sides. Results for the left and right side of the body for all strides and isolated transitions are practically the same, with the results of the centre position just slightly below them. The same applies for accuracy, with small mean average error within the bounds of one sample, but with a higher dispersion of errors by transitions. It is interesting that similar results are obtained for measurements on different body parts. Slightly poorer performance is shown for transitions, especially on the positions that are higher on the body. This is also obvious from the accuracy results. This can be explained by the fact that higher body positions have a lower influence on gait dynamics, and consequentially less discriminative stride patterns collected by sensors attached to these positions. Concerning overall accuracy of stride time extraction, it can be seen in Figure 7a,b that accurate estimation of stride times obtained from the proposed approach on smartphone data is confirmed by the correlation analysis and Bland–Altman plot, where both show good matching between detected and reference stride times. Regardless of the fact that a sampling frequency of 25 Hz was used for the evaluation of the approach, the difference between the stride times is very close to zero, with errors falling within two samples with a standard deviation of 1.96.

The proposed approach validated on the eGait dataset reveals robust and accurate performance on inertial data acquired from shoe sensors. First, it should be emphasized that these results were obtained based on the assumption that just the noise threshold is enough for efficient stride extraction. In contrast with smartphone data, where different positions convey different signal shapes and dynamics, inertial data collected from shoe-mounted sensors conveys stable dynamics. Therefore, the stride segmentation procedure can be performed by solely using a noise threshold that is directly related to a component or sensor (i.e., acceleration noise) and $v_{i}$ does not need to be considered. In general, the results of the evaluation are very promising, since shoe-mounted sensors produce very clear and quasi-repetitive stride patterns, and the proposed approach is very efficient on such data. This is confirmed by the overall efficiency of stride segmentation of over 98%, with stride detection bias within one sample varying by approximately ±1 sample at a sampling frequency of 102.4 Hz. The MAE is under one sample, with 95% of errors falling within the window of ±2 samples. Consequentially, there is also very accurate matching between estimated stride times with the ground truth, as shown by the regression analysis and the Bland–Altman plot in Figure 9. Furthermore, if we directly compare the obtained results with similar recent approaches on the same dataset (Figure 10), we can see that the proposed approach slightly outperforms the approaches published in [10,36], where the following important facts that speak in favor of our approach should be considered: (1) our approach does not need any templates, and segmentation can be efficiently performed by simply using noise threshold; (2) it uses same setup for both controlled and free walk; and (3) unlike [10], where an optimal combination of components is used per cohort, the proposed approach uses a single combination of estimators and components for all cohorts.

Evaluation of gait event extraction by using the proposed approach revealed very good matching with the eGait system [18] that represents a known and accurate reference. Each of three detected gait events (toe-off, heel-strike, and mid-stance) were examined separately. Concerning TO events, results reveal practically perfect matching with the eGait system with near-zero errors, as can be seen in Figure 11a and Figure 12a. Detection of toe-off events is the least problematic since these events are defined as zero-crossings on the gyroscope *z*-axis that are clearly distinguishable in most cases. In contrast, heel strike events turn out to be most difficult to detect. Namely, the decision function anticipates an abrupt spike in the negative direction in acceleration on the *y*-axis, that is in most cases somehow blurred within the signal. This is mostly the case in transitions. Thus, the proposed approach resulted in decreased detection coverage, especially during free walks that contain many transitions. However, detection accuracy is still high and with minimal difference compared to the eGait system. In order to increase the coverage, one way would be to redefine the decision function for a gait event on account of slightly reduced detection accuracy. In contrast with heel-strike events, mid-stance events resulted in very good coverage, but decreased accuracy when compared to the eGait system. The problem is in the non-strict definition of a mid-stance gait event, which is represented by a quite broad and flat zero-valued plateau in the gyroscope signal that represents zero-velocity time instants. These are crucial for further processing algorithms (i.e., stride length estimation). However, decreased accuracy compared with the eGait system falls within a few 10 ms. That means that detected mid-stance gait events are detected within the plateau and are accurate enough to produce a comparable functionality with zero velocity-based gait assessment algorithms. Furthermore, when comparing the results with the eGait system based on experiment type and between all three cohorts, it can be concluded that the consistency of these results further proves the universality of the proposed approach.

The results also show that the proposed approach shows considerable reliability and self adaptability based on local cyclicity, and thus also copes well with transitions. However, the approach might have some issues with isolated strides as it expects at least two consecutive strides within a stride sequence. Besides that, detection of the onsets of transitions might be challenging. The approach also assumes that stride onsets and terminations fall in the exact same location, which is most likely during natural walking. This was also obvious in the experimental datasets, i.e., eGait [10] for elderly and PD patients. Furthermore, the proposed approach also showed good performance in situations where components and corresponding fiducial points were not defined in advance (i.e., by application of accurate step detection on smartphones). It is true that in a controlled environment (i.e., shoe-mounted sensors) the approach reveals even more accurate results, but operability of the approach on data collected in uncontrollable scenarios is also very efficient. Thorough evaluation of the proposed approach revealed comparable or even improved results when compared with the latest state-of-the-art (i.e., [10,26]), and even with commercially available solutions [14], thus proving the universality of the proposed approach.

## 6. Conclusions

We have presented a robust, efficient, and accurate approach for stride segmentation of inertial signals based on local cyclicity estimation and sensor fusion, which is also capable of stride sequence extraction and gait event detection. We have shown that the proposed approach is able to operate with different combinations of local cyclicity estimators and components. Besides two optional parameters, the stride reliability threshold $h_{v}$ used in uncontrolled conditions and the noise variability threshold $h_{r}$ that depends on a used sensor type, the approach also does not rely on any templates or supervision. Due to its local nature of operability, it is also efficient for transitions. Due to its robustness, the proposed approach can be directly used for reliable assessment of temporal gait parameters, including stride time, cadence, etc. The design of the approach also allows the implementation and operability in real time, i.e., on-the-fly combined with other gait assessment approaches.

Thus, we believe that the advantages of the proposed approach and scientific contributions exposed in light of its evaluation show good potential for wide applicability in the corresponding scientific communities. The presented approach has already been implemented and operates in our real-time prototype platform for gait recognition based on [3] as a module for gait sequence extraction and stride segmentation. Our further research activities will also rely on the proposed approach, especially in the fields of long-term gait monitoring and development of novel algorithms for assessment of spatio-temporal gait parameters in uncontrollable scenarios, as well as advanced semi-supervised smart annotation for movement activity data.
